# Differential kinase activity of ACVR1 G328V and R206H mutations with implications to possible TβRI cross-talk in diffuse intrinsic pontine glioma

**DOI:** 10.1038/s41598-020-63061-0

**Published:** 2020-04-09

**Authors:** Hongnan Cao, Miao Jin, Mu Gao, Hongyi Zhou, Yizhi Jane Tao, Jeffrey Skolnick

**Affiliations:** 1 Center for the Study of Systems Biology, School of Biological Sciences, Georgia Institute of Technology, 950 Atlantic Drive, NW, Atlanta, Georgia 30332 United States; 2 Department of BioSciences, Rice University, Houston, Texas 77005 United States

**Keywords:** Kinases, CNS cancer, Target identification, CNS cancer

## Abstract

Diffuse intrinsic pontine glioma (DIPG) is a lethal pediatric brain cancer whose median survival time is under one year. The possible roles of the two most common DIPG associated cytoplasmic ACVR1 receptor kinase domain mutants, G328V and R206H, are reexamined in the context of new biochemical results regarding their intrinsic relative ATPase activities. At 37 °C, the G328V mutant displays a 1.8-fold increase in intrinsic kinase activity over wild-type, whereas the R206H mutant shows similar activity. The higher G328V mutant intrinsic kinase activity is consistent with the statistically significant longer overall survival times of DIPG patients harboring ACVR1 G328V tumors. Based on the potential cross-talk between ACVR1 and TβRI pathways and known and predicted off-targets of ACVR1 inhibitors, we further validated the inhibition effects of several TβRI inhibitors on ACVR1 wild-type and G328V mutant patient tumor derived DIPG cell lines at 20–50 µM doses. SU-DIPG-IV cells harboring the histone H3.1K27M and activating ACVR1 G328V mutations appeared to be less susceptible to TβRI inhibition than SF8628 cells harboring the H3.3K27M mutation and wild-type ACVR1. Thus, inhibition of hidden oncogenic signaling pathways in DIPG such as TβRI that are not limited to ACVR1 itself may provide alternative entry points for DIPG therapeutics.

## Introduction

Diffuse intrinsic pontine glioma (DIPG) is an aggressive brainstem pediatric cancer with no effective treatment and a median survival of less than a year according to the International DIPG Registry^1^. Clinical DIGP tumors harbor a repertoire of somatic mutations including the most frequent (~80%) histone H3.3 or H3.1 K27M variants^2–5^. Spatiotemporal genomic mapping of DIPG from whole brain autopsies suggested that the H3K27M histone mutations are often paired with either ACVR1 kinase and/or tumor suppressor TP53 mutations in tumor initiation, followed by later oncogenic alterations often in the phosphoinositide 3-kinases (PI3K) pathway in subclones^4^. Epigenetic profiling of DIPG using chromatin immunoprecipitation sequencing demonstrated a genome-wide distribution of H3K27M that is highly correlated with active transcription as indicated by colocalization with H3K27ac (acetylation) and RNA polymerase II^6^. H3K27M is typically excluded from genomic regions marked with H3K27me3 (trimethylation) where the histone methyltransferase polycomb repressive complex 2 (PRC2) resides^6^. Both spatiotemporal immunostaining and epigenetic mapping of transcriptional dependencies of DIPG traced the tumor origin to be oligodendroglial precursor cells expressing markers of an undifferentiated neural cell state^7,8^.

The understanding of the genetic and epigenetics context of DIPG led to therapeutic efforts that targeted chromatin modifiers such as histone deacetylase (HDAC), demethylase or methyltransferase complexes^6,8–11^ as well as kinases such as ACVR1 (also called ALK2)^2,12^. These studies yielded promising preclinical efficacy of single agent or combinations in different DIPG cell and mice xenograft models. However, no effective treatment for DIPG patients exists^1^, despite numerous clinical trials including a pilot precision medicine trial for DIPG^13^. This failure might be due to: (1) incomplete understanding of the fundamental oncogenesis, relapse and drug resistance mechanisms of DIPG and/or (2) pleiotropic effects of epigenetic interventions that are context-dependent in both DIPG and normal physiology.

This study was motivated by the observations of the presence in DIPG patients of ACVR1 heterozygous mutations (~25% frequency) that are strongly correlated with the H3.1K27M mutation^5,14,15^ and the contrasting prognosis statistics showing either the same or longer survival time of DIPG patients harboring tumors with ACVR1 mutations^2,16^. Indeed, a very recent genomics meta-analysis of clinical DIPG data (n = 212) indicated that the beneficial clinical outcome of ACVR1 mutations (50/212) is largely restricted to the G328E/V/W subgroup (median survival of 16.0 months, patients number n = 28). In contrast, changes in survival time are statistically insignificant for the R206H subgroup (13.0 months, n = 13) when compared with ACVR1 wild-type patients (10.0 months, n = 162)^2^. The same study showed that the ACVR1 mutant DIPG patient population is enriched in both long-term survivors (>24.0 months, 5/8) and top 10% survivors (>19.0 months, 10/21), all of which harbor G328E/V/W mutant tumors^2^. These clinical prognosis statistics within the DIPG patient population argue against targeting these ACVR1 mutants to treat DIPG. In principle, while ACVR1 might be an attractive target for kinase inhibitors, is this a viable strategy for DIPG? As detailed below, our results suggest that further target validation is needed and may lead to the discovery of alternative therapeutic strategies.

## Results

### Intrinsic kinase activity of purified wild-type, R206H and G328V ACVR1 kinase domains

**Figure 1:**
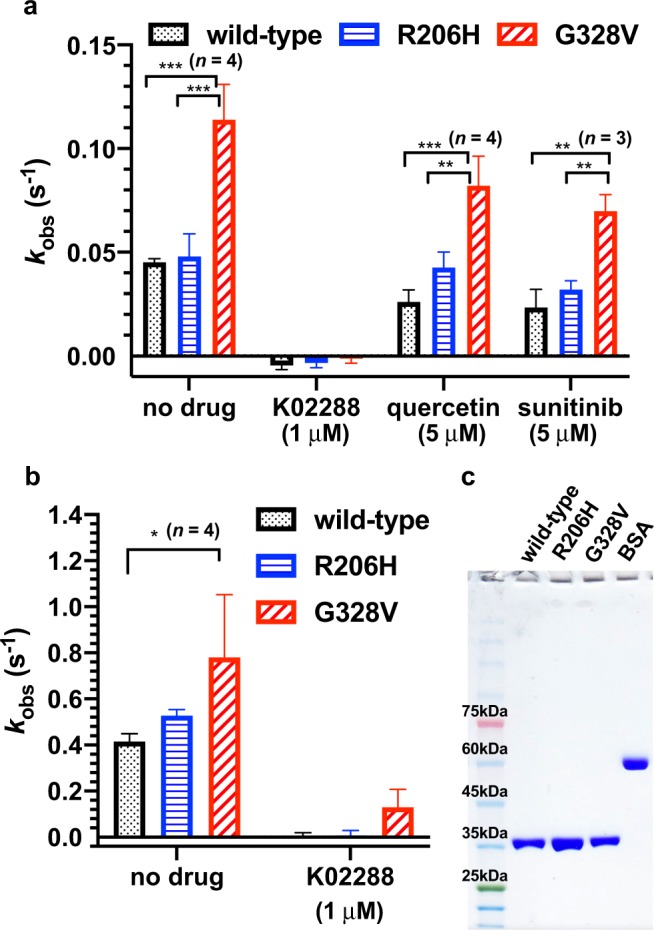
The intrinsic ATPase activity wild-type, R206H and G328V mutants of ACVR1 kinase domains with 0.5 mM ATP in pH 7.3 kinase buffer. (**a**) 22 ^o^C and (**b**) 37 ^o^C. The turnover rate *k*_obs_ is quantified based on the ADP produced and normalized by the native ACVR1 concentration (see Methods). *p < 0.05, **p < 0.01, ***p < 0.001, unpaired *t*-test. (**c**) Coomassie stained SDS-PAGE gel of purified ACVR1 kinase domain stocks used for kinase assays. See Fig. S5 for the full-length gel image.

To assess the molecular modes of actions of ACVR1 mutations in DIPG with apparently different prognoses, we measured the intrinsic kinase activity of the G328V and R206H mutants by quantifying the ATP hydrolysis rate of the ACVR1 cytoplasmic kinase domain lacking the adjacent N-terminal GS regulatory loop in order to isolate truly ligand-independent basal activity. The kinase domains were recombinantly expressed in and purified from SF9 insect cells (Fig. 1c). The steady-state rate *k*_obs_ measured under saturated ATP conditions is comparable to the rate constant *k*_cat_. The concentration of 0.5 mM ATP (physiological level^17^ ~3 mM) used here is estimated to saturate ACVR1 based on the reported Michaelis constant *K*_m_ from radiolabeled ATP kinase assays at room temperature for wild-type (16 μM) and disease mutants (35–40 μM) L196P, Q207E, R258S, G328E within or near the GS domain^18^. Similar ATP or ADP affinity (*K*_d_) between wild-type and the R206H mutant is also suggested based on comparable thermal shifts (ΔT_m_), while the G328V mutant displays a slightly higher ΔT_m_ indicative of higher nucleotide affinity (smaller *K*_d_). (Supplementary Fig. 1). All the observed basal activities of wild-type, R206H and G328V can be abolished by a known ACVR1 inhibitor K02288 (IC_50_ of 1–2 nM; *K*_d_ of 7.9 nM)^19^ at 1 μM (Fig. 1). We also observed partial inhibition of ACVR1 wild-type and mutants by typical promiscuous kinase inhibitors such as the natural flavonoid quercetin and the FDA-approved chemotherapy drug sunitinib at 5 μM, consistent with their weaker affinity as measured by thermal shift assays (2.2 and 0.93 μM) (Supplementary Fig. 2).

We observed a 2.5 fold increase in the intrinsic kinase activity of the G328V mutant over the wild-type ACVR1 kinase domain at 22 ^o^C and a 1.8 fold increase at 37 ^o^C (Fig. 1a,b). On the other hand, the R206H mutant kinase domain shows basal kinase activity that is indistinguishable from wild-type (Fig. 1a,b). Thus, ACVR1 kinase activity (*k*_cat_^G328V^ > *k*_cat_^R206H^ ~ *k*_cat_^wild-type^) is consistent with the reported median survival time *t*_1/2_ of DIPG patients^4^ (*t*_1/2_^G328E/V/W^ > *t*_1/2_^R206H^ ~ *t*_1/2_^wild-type^). Whether this is a driver of patient survival is unclear.

We further note that DIPG patients harboring the R206H mutation show a median survival time that is statistically indistinguishable from the wild-type subgroup^2^. The R206H mutation switches ACVR1 from FKBP12 inhibition to type-II receptor-dependent activation via GS domain phosphorylation and disrupting protein-protein interactions^16,20–24^. This effect may play a minor role, if any, in DIPG progression. A supporting observation is that while the R206H mutation drives a rare inherited connective tissue disorder fibrodysplasia ossificans progressiva (FOP)^25^, no DIPG was reported for FOP patients.

### Structural basis for increased intrinsic kinase activity of G328V mutant vs. wild-type ACVR1

**Figure 2:**
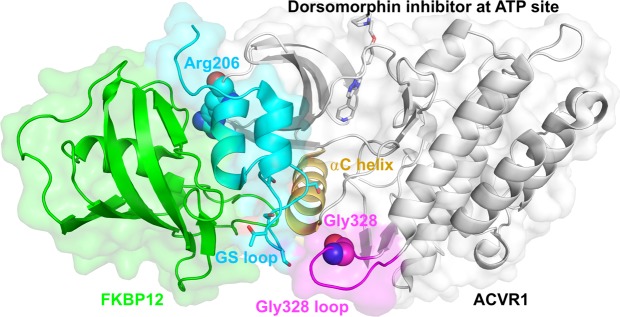
Locations of the Gly328, Arg206, Gly328 loop (magenta), GS domain (cyan) and αC helix (golden) allosteric hub in ACVR1 (gray elsewhere) in complex with FKBP12 (green, left) (PDB: 3H9R)^20,22^. The backbone is shown as ribbons, Gly328 and Arg206 as spheres, dorsomorphin at the ATP site and the conserved Thr/Ser phosphorylation sites on the GS loop^20,22^ as sticks.

As suggested by Fig. 2, the increased intrinsic kinase activity of the G328V ACVR1 mutant may be due to the change in tilt of the conserved αC helix on one end leading to active conformation of the ATP site on the other end^16,20,22^. R206H uses the same allosteric hub αC helix to confer activation, but does so remotely by unlocking the GS loop from the αC helix via disruption of FKBP12 inhibition^16,20,22^. Activating phosphorylation of TβRI (ALK5) by TβRII is also suggested to disrupt GS loop and αC helix interactions^22,23^. Interestingly, the GS loop and Gly328 loop each contact the C-terminal tip of the αC helix on opposite sides indicating they act as separate structural elements to modulate ACVR1 activation. This may be the key molecular basis underlying the biochemical differences of these ACVR1 mutations.

### Thermal stability of purified wild-type, R206H and G328V ACVR1 kinase domains

**Figure 3:**
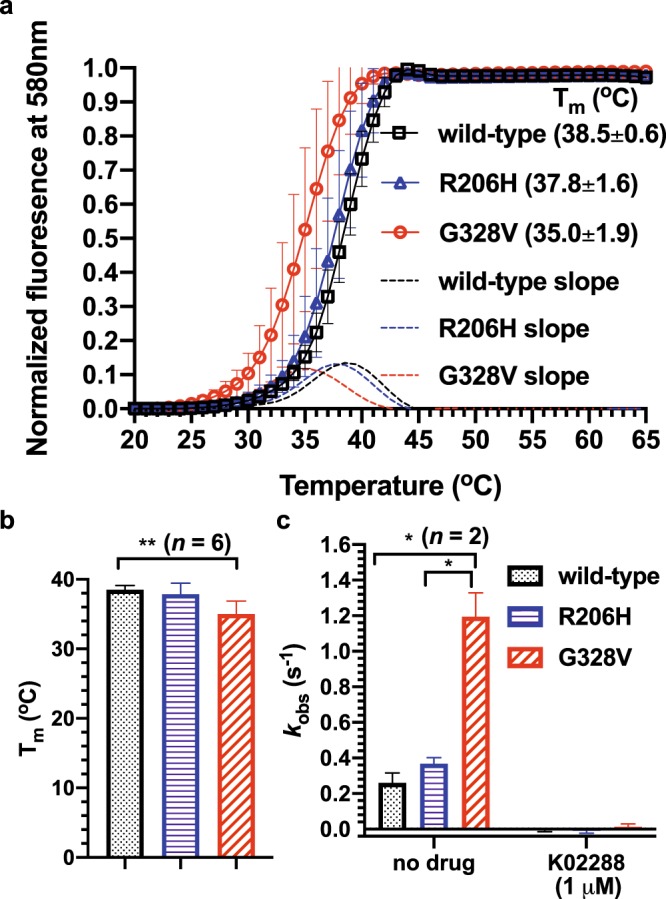
Thermal stability of purified ACVR1 kinase domains. (**a**) Melting curves of wild-type, R206H and G328V ACVR1 kinase domains. (**b**) Plot of T_m_ values defined as the temperature at maximum slope of each curve. **p = 0.0015, unpaired *t*-test. (**c**) The intrinsic ATPase activity wild-type, R206H and G328V mutants of ACVR1 kinase domains with 0.5 mM ATP and 0.5 mg/ml dephosphorylated casein substrate in pH 7.3 kinase buffer at 37 ^o^C normalized by the native ACVR1 concentrations.

The G328V but not the R206H ACVR1 kinase domain shows a negative thermal shift ΔT_m_ of −3.5 ^o^C compared to wild-type, suggesting that the G328V mutant is thermodynamically less stable (Fig. 3a,b). This instability explains the apparent reduced activity at 37 ^o^C for the G328V kinase domain compared to wild-type when it is normalized by the total enzyme population (Supplementary Fig. 3a) as opposed to when it is corrected for the native population (Fig. 1b). We note that the few reported kinase assays using purified ACVR1 (residues 201–499 as here) were performed at room temperature^18,19,26^. In previous work, the percentage inhibition, rather than the absolute rate was often measured to assess drug potency or selectivity^18,19,26^. Whether the thermal stability of a kinase domain affects full-length ACVR1 function under physiological conditions is unknown. Nonetheless, the increased intrinsic kinase activity of G328V but not the R206H ACVR1 kinase domain at 22 ^o^C (Fig. 1a) and at 37 ^o^C (Figs. 1b, 3c, Supplementary Fig. 3b), agrees with previous phenotypic studies using ACVR1 transfected DIPG and FOP mouse cell models to compare signaling activities of different ACVR1 mutants and wild-type^12,25^. These studies showed that G328V expression caused higher Smad/1/5/8 phosphorylation and downstream target gene ID1 expression levels in the DIPG cell model or downstream luciferase reporter gene expression levels in the FOP cell model than R206H and wild-type, either constitutively or upon specific extracellular stimulations such as Activin A^12,25^.

### Implications of Off-targets of ACVR1 inhibitors to the TβRI pathway in DIPG Therapeutics

Although there clearly exists a high frequency of ACVR1 somatic mutations in DIPG patients^5,14,15^ and the demonstrated preclinical potency of ACVR1 inhibitors at a µM level to inhibit DIPG cells with different biological backgounds^2,12^, we suggest that additional target validation and mechanistic studies of ACVR1 and associated pathways for DIPG therapeutics are required for the following reasons: First, the G328E/V/W mutations correlate with longer patient survival^2,15^. At least for the G328V mutation, its better prognosis might be due to increased kinase activity (Fig. 1). Moreover, G328V is the only ACVR1 mutation unique for DIPG that is not found in FOP or heart diseases^25^. Second, the preclinical efficacy^2,12^ of ACVR1 inhibitors LDN193189, LDN214117 and LDN212854 in DIPG cell and mouse xenograft models may result from off-target effects. This might include inhibition of the previously experimentally verified *in vitro* off-targets^18,19,27^ ABL1, PDGFR and MAP4K4 kinases. This is consistent with the expression of these off-targets in human gliomas according to the Human Protein Atlas (proteinatlas.org)^28^. Application of the FINDSITE^comb2.0^ virtual target screening^29^ algorithm against the human proteome predicts additional off-targets for pre-clinical ACVR1 inhibitors LDN193189, LDN214117, LDN212854 and K02288 involving type-II TGF-β family receptors such as TβRII (Supplementary Table 1). Type-II receptors are responsible for phosphorylation and activation of cognate type-I receptors. We note that this off-targeting situation is not unprecedented. For example, a recent CRISPR-Cas9 mutagenesis-based mode-of-action study demonstrated that off-targeting dominated cancer drug efficacy in clinical trials, whereas the putative primary target was actually not the cancer driver at all^30^.

**Figure 4:**
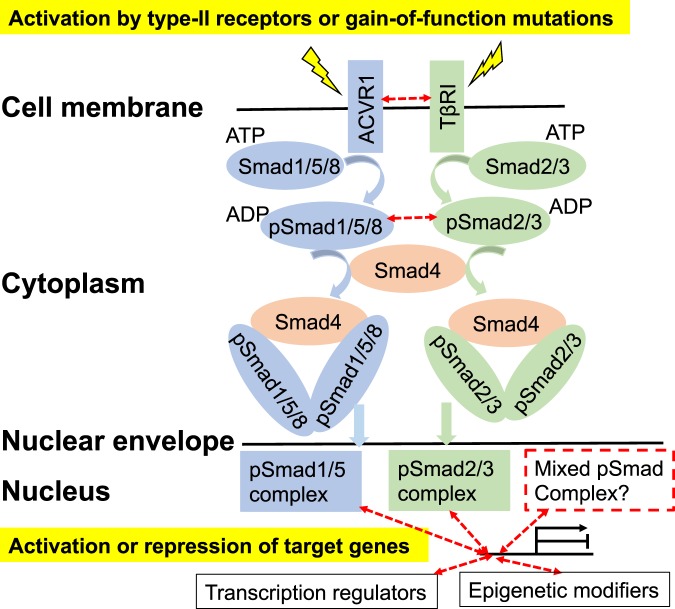
Cross-talk between ACVR1 and TβRI pathways proposed in DIPG. Dashed red arrows indicate cross-talk inferred from known physical interactions. Smad1/5/8 and Smad2/3 upon phosphorylation (by ACVR1 and TβRI, respectively) bind Smad4 to form different complexes, enter the nucleus and regulate transcription^31,32^. ACVR1 can form a receptor heterocomplex with TβRI^48^. Heterocomplexes between phosphorylated Smad1/5 and Smad2/3 were also reported before^48–50^. Antagonism of Smad1/5 and Smad2/3 associated pathways was reported in the contexts of *Xenopus* embryogenesis^51^, endothelial^49^, keratinocyte^50^, myoblast^50^, and human breast cancer^50^ but not mouse mammary epithelial cells^48^.

Can the knowledge of better prognosis of activating ACVR1 mutations help design of effective DIPG therapeutics? We conjecture that the increased kinase activity of the ACVR1 G328V mutation interferes with essential drivers of cancer progression *in vivo*. If confirmed, this implies that targeting a DIPG essential pathway affected by ACVR1 not limited to ACVR1 itself might be a plausible therapeutic strategy post-DIPG diagnosis. The rationale is that at the time of intervention, tumors harboring somatic mutations have already occurred and the mutational dependence of cancer progression may drift from that responsible for cancer onset. One possible candidate for DIPG intervention is the TβRI pathway. ACVR1 can physically cross-talk to TβRI at both the receptor and/or Smad levels in several types of human cancers (Fig. 4) despite little knowledge of their antagonism or synergy in the DIPG context. The contextual roles of TGF-β and TβRI signaling are well established as tumor suppressive^31,32^ in normal tissues vs. proliferative, metastatic or immunosuppressive^31–33^ during cancer progression.

Interestingly, we note that there is a marked decrease of gene transcription of TβRII, TβRI, TGF-β1 (extracellular agonist of the TβRII and TβRI heterocomplex) and Smad3 during the mid-fetal period of normal brain development coinciding with the transcription peak of ACVR1 discovered by analyzing the human brain transcriptome (hbatlas.org)^34^ (Supplementary Fig. 4). The onset of DIPG was suggested to occur in this period based on overlapped histone H3K27M expression peaks^2^. In contrast to normal brain development, not only ACVR1 but also TβRI type-I receptor was reported to be overexpressed in the primary DIPG tumors vs. unaffected normal brain tissues based on RNA sequencing of a cohort of DIPG patients representing different types of tumor mutational burden^10^. It is possible that in DIPG tumors, unlike normal brain development, TβRI signaling is amplified to drive cancer progression at the post-diagnosis stage that is most relevant for DIPG therapeutics. Moreover, the effector Smad proteins that are phosphorylated and activated by type-I TGFβ family receptors such as TβRI and ACVR1 are known to play essential roles in global regulation of gene expression at the levels of transcription regulation, epigenetic remodeling, RNA splicing, miRNA processing, m6A mRNA methylation^31,35^. Mechanistically, the interplay between ACVR1 and TβRI in Smad utilization may provide additional control besides histone mutations to shape the epigenetic landscape^35,36^, expression profile and predisposition to secondary subclonal mutations^37^, and consequently determine the DIPG cell states and clinical outcomes.

**Figure 5:**
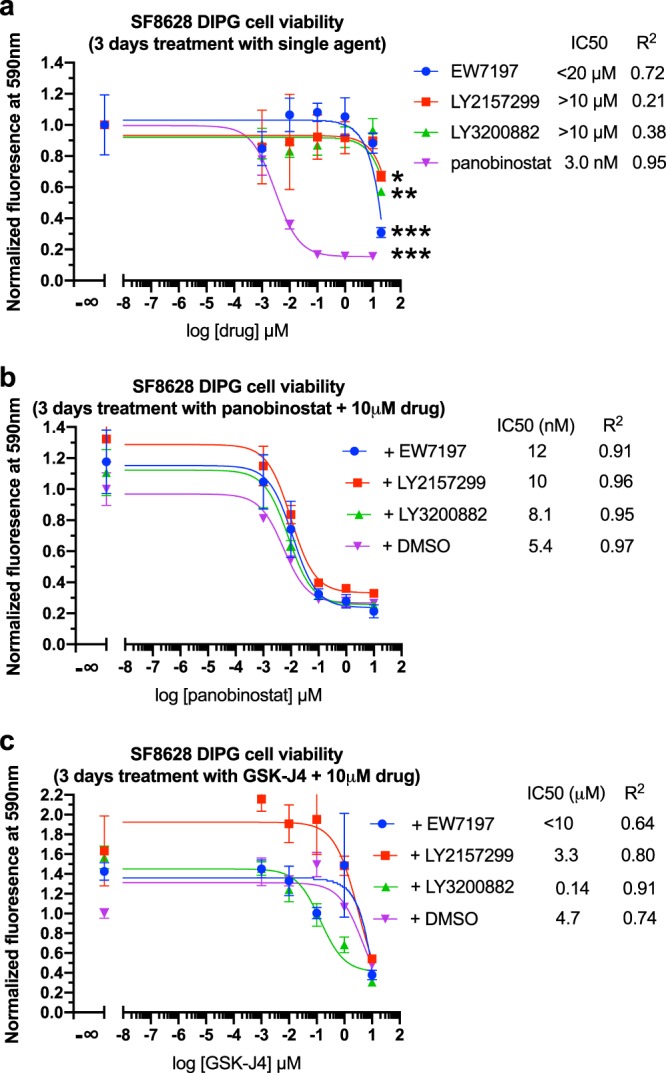
Dose response of SF8628 DIPG cell viability. (**a**) Single agent study of TβRI inhibitors. *p < 0.05, **p < 0.01, ***p < 0.001, *n* = 4, unpaired *t*-test of highest dose vs. control. (**b**) panobinostat and (c) GSK-J4 tested in combination with 10 µM TβRI inhibitors. The relative cell viability of drug(s) treated samples over the DMSO control is shown. When, as in (**c**) for EW7197, the IC50 value is inconclusive from curve fitting, an apparent upper limit is estimated.

**Figure 6:**
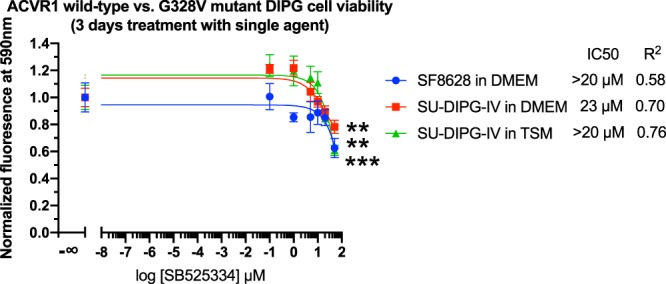
Dose responses of ACVR1 wild-type and G328V mutant DIPG cell viability to the treatment of SB525334, a selective inhibitor for TβRI over ACVR1. (**a**,**b**) Single agent study of TβRI inhibitors. **p < 0.01, ***p < 0.001, *n* = 4, unpaired *t*-test of highest dose vs. control. The genotypes for patient tumor derived DIPG cell lines are ACVR1 wild-type and histone H3.1K27M for SF8628 and ACVR1 G328V mutant and histone H3.3K27M for SU-DIPG-IV. DMEM (the default FBS-containing media for SF8628 used by manufacture and previous studies^12^) and TSM (a serum-free multi-growth factors-containing complex Tumor Stem Medium derived from previous studies on SU-DIPG-IV^2,12^) are different media culture conditions under examination (see methods).

**Figure 7:**
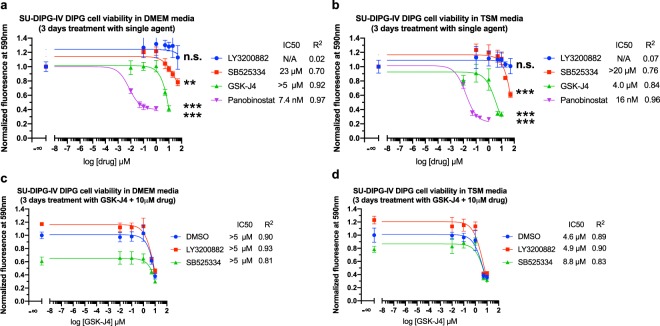
Dose response of SU-DIPG-IV cell viability. (**a**,**b**) Single agent study of TβRI inhibitors. **p < 0.01, ***p < 0.001, n.s. *p* > 0.05, *n* = 4, unpaired *t*-test of highest dose vs. control. (**c**,**d**) GSK-J4 tested in combination with 10 µM TβRI inhibitors. The relative cell viability of drug(s) treated samples over the DMSO control is shown. SU-DIPG-IV Cells were cultured and tested in DMEM (**a**,**c**) and TSM (b), (d) media, respectively (see methods).

On this basis, using Tox-8 cell viability assays^38^, we explored the potential of TGF-β signaling blockers^39^ to inhibit DIPG growth. We found that single agent treatment of TβRI inhibitors EW7197 (vactosertib), LY3200882 and LY2157299 (galunisertib) at a 20 µM dose showed a statistically significant inhibitory effect on the growth of patient derived SF8628 DIPG cell line harboring the H3.3K27M mutation and wild-type ACVR1 (Fig. 5a). We further showed that an investigational TβRI blocker SB525334, with a previously reported ~1000-fold selectivity for TβRI over ACVR1^40^, demonstrated statistically significant inhibition in both SF8628 (ACVR1 wild-type, histone H3.3K27M) and SU-DIPG-IV (ACVR1 G328V, histone H3.1K27M) DIPG cells at a relatively high 50 µM dose (Fig. 6). In contrast, LY3200882 inhibits SF8628 DIPG cells at a 20 µM dose (Fig. 5a) but not SU-DIPG-IV cells (Fig. 7a,b), suggesting lower sensitivity of the latter to TβRI blocking.

As positive controls, the epigenetic drugs histone demethylase inhibitor GSKJ-4 and the histone deacetylase (HDAC) inhibitor panobinostat inhibited the growth of both SF8628 and SU-DIPG-IV cell lines to a similar extent (Figs. 5a and 7a,b). Consistent with previous reports^10,11^, panobinostat (nM IC50) is more potent than GSK-J4 (µM IC50). At 10 µM, LY3200882 exhibited a synergistic effect with GSK-J4 (Fig. 5c) and lowered the apparent half maximum inhibition concentration IC50 value of GSK-J4 (Fig. 5c) by over 10-fold in the SF8628 cell model. No synergistic effect of TβRI blockers with panobinostat on SF8628 inhibition was observed (Fig. 5b). Neither single agent inhibition by LY3200882 alone nor synergy with GSK-J4 was observed in the SU-DIPG-IV cell model harboring the activating ACVR1 G328V and H3.1K27M mutations (Fig. 7). This suggests that the constitutively activating ACVR1 G328V mutation may potentially suppress TβRI pathway signaling and diminish the DIPG inhibition effect of TβRI inhibitors. Overall, similar inhibition profiles of drug treatments in SU-DIPG-IV cells were observed under DMEM (the same was used to culture SF8628 commercial cell line) and TSM media culture conditions (derived from previously reported SU-DIPG-IV studies)^12^ (Figs. 6 and 7). This is informative for future DIPG drug discovery studies on the choice of culture conditions; an issue that was not thoroughly explored prior to our study. Taken together, the results on the inhibition effects of TβRI blockers on ACVR1 wild-type and G328V mutant human DIPG cells are generally consistent with our hypothesis of potential cross-talk between ACVR1 and TβRI pathways in DIPG.

## Discussion

**Figure 8:**
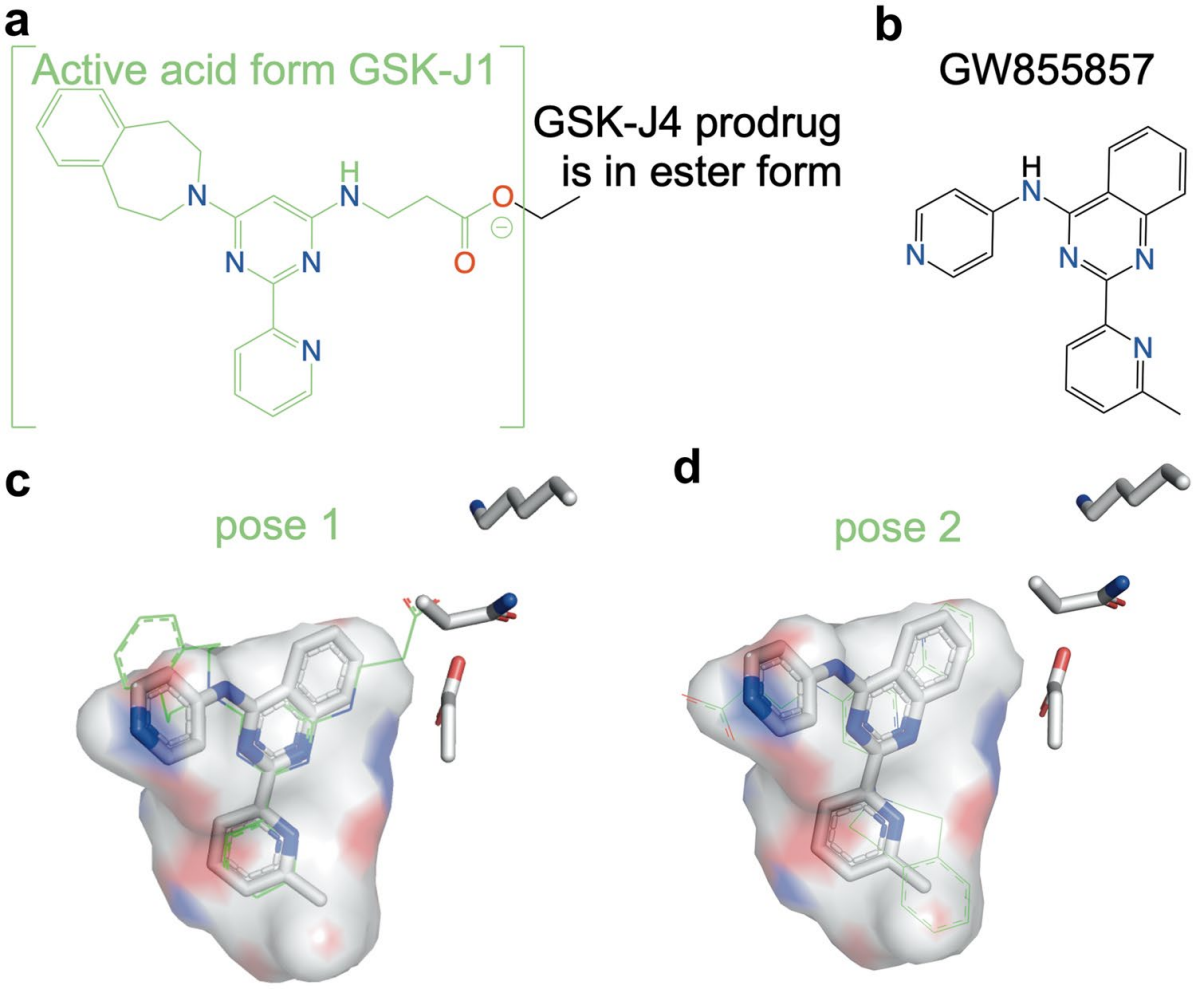
Structural similarity of the GSK-J4 and the TβRI inhibitor GW855857^41^ suggests that GSK-J4 off-targeting TβRI predicted by FINDSITE^comb2.0^ is plausible. Their respective chemical structures are shown in (**a**) GSK-J4 and (**b**) GW855857. When GSK-J4 in the ester form is turned into the active acid form GSK-J1, the latter inhibits H3K27 histone demethylase^52^. GW855857 inhibits TβRI (ALK5)^41^. Using the PyMOL Molecular Graphics System Version 2.3.2 (Schrödinger, LLC), the GSK-J1 ligand structure (from PDB: 4ASK)^52^ was manually docked into the GW855857 binding pocket in its inhibitory complex crystal structure with TβRI (PDB: 3HMM)^41^. (**c**) Pose 1 of GSK-J4 (thin sticks, carbon in green) is generated based on spatially matching its common scaffold with GW855857 (thick sticks, carbon in white). Pose 1 allows for potential polar interactions between the carboxylic group of the docked GSK-J4 and the TβRI residues near the solvent exposed pocket entrance. (**d**) Pose 2 is rotated approximately 120 ^o^C within the ligand plane from Pose 1 and appears to allow for a better steric fit in the pocket.

Applying the FINDSITE^comb2.0^ virtual target screening method^29^ we predicted an unexpected drug-protein interaction between GSK-J4 and TβRI. This prediction is supported by the structural similarity between GSK-J4 and the experimentally verified TβRI inhibitor GW855857 present in an inhibitory complex structure with TβRI (PDB: 3HMM)^41^ (Fig. 8). GSK-J4 appears to be sterically accommodated in the inhibitory pocket of TβRI either the same way as GW55857 or with a rotated pose relative to that of GW55857. This potential off-targeting may partially explain the observed synergy between GSK-J4 and the TβRI inhibitor LY3200882 in SF8628 DIPG cells growth inhibition (Fig. 5c). But the actual cause of this interesting and possibly clinically relevant synergy, needs further elucidation. Note that not all TβRI inhibitors show synergistic effects with GSK-J4 (Fig. 5c). Considering the quality of the curve fitting and experimental errors, we estimate that GSK-J4 has an IC50 < 10 µM when combined with 10 µM EW-7197, which is comparable to IC50 ~4.7 µM for the GSK-J4 alone control (plus DMSO) (Fig. 5c).

It should be noted that previous studies showed that both panobinostat and GSK-J4 restore H3K27 trimethylation epigenetic marks in DIPG cells^10,11^. However, the differential gene expression profiles (drug vs. control) of these two epigenetic drugs reported in these studies don’t appear to resemble each other. Nonetheless, one study^10^ showed a synergistic effect on inhibiting DIPG cells between panobinostat and GSK-J4. This underscores the limited understanding of the molecular mechanisms that link epigenetics to gene transcription and phenotypical response in DIPG. During the revision of our manuscript, a new study on high-throughput combination drug screening against DIPG showed synergy between panobinostat and the proteasome inhibitor marizomib on inhibiting patient derived DIPG cells^42^. That work demonstrated a modest benefit of the combination therapy with a ~20% increase in overall survival time of xenograft mice models^42^. Interestingly, the same study also showed varied combination effects between panobinostat and different CDK inhibitors^42^, i.e., they were synergistic, antagonistic or essentially absent depending on the individual CDK inhibitor. CDK2/4 kinases are known to regulate the subcellular localization, stability and function of Smad proteins downstream of TβRI via direct phosphorylation^31^. This is consistent with our findings above that no synergistic effects were observed between panobinostat and TβRI inhibitors (Fig. 5b).

As highlighted by the failure to prolong patient survival of a recent pilot precision medicine trial for children with DIPG^13^, there is a pressing need to develop alternative approaches. To address this crucial issue, we examined whether ACVR1 is likely to be a promising target for DIPG therapy. We demonstrated that DIPG associated G328V mutant showed increased kinase activity, while the R206H mutant showed the same activity as the wild type ACVR1. We then conjectured that the increased kinase activity of the G328V mutant possibly underlies the longer survival times of these patients. The question then arose as to what might cause this enhanced survival rate due to increased kinase activity? Since ACVR1 is known to cross-talk with the TβRI pathway, TβRI inhibition might be one possibility. To explore whether this conjecture was true, we examined whether TβRI inhibitors could inhibit DIPG cell line growth. Our experiments confirmed the inhibition effects of several TβRI inhibitors on ACVR1 wild-type and G328V mutant patient tumor derived DIPG cell lines at 20–50 µM doses. SU-DIPG-IV cells harboring the histone H3.1K27M and activating ACVR1 G328V mutations appeared to be less susceptible to TβRI inhibition than SF8628 cells harboring the H3.3K27M mutation and wild-type ACVR1. There are a number of broader implications of this result well. Since the TGFβ pathway promotes tumor progression in the context of *in vivo* tumor microenvironments by suppressing and evading the immune system^39^, immune checkpoint inhibitors might be relevant for DIPG. These issues will be explored in future work.

## Methods

### Protein expression and purification from insect cells

DNA sequences of human wild-type ACVR1, R206H and G328V mutant kinase domains (containing codons for an N-linked 6xHis tag and residues 201–499 of the Uniprot sequence ID: Q04771) each in a pFastBac1 vector were synthesized by Genscript, USA. The codons were optimized by Genscript for protein expression in insect cells. The commercial Bac-to-Bac baculovirus expression system (Life Technologies, USA) was used to express the human wild-type ACVR1, R206H and G328V mutant kinase domains in SF9 insect cells. SF9 cells were maintained as shaker flask cultures at 27°C with a constant shaking speed of 125 rpm/min in Sf-900TM II SFM medium (Gibco, USA). Insect cells at a density of ~2.5 × 10^6^ cells/ml were infected with 10% P2 viral stock and harvested after 48 hours post-infection by centrifugation at 600xg for 15 minutes. Pelleted cells were resuspended in a lysis buffer (300 mM NaCl, 50 mM Tris-HCl pH 8.0, 10% glycerol, 1 mM NaN_3_, 5 mM 2-Mercaptoethanol) and lysed by sonication on ice. The lysate was clarified by centrifugation at 48,000xg for 45 minutes. The supernatant fraction was then incubated with pre-equilibrated Ni-NTA resin (Thermo Fisher Scientific, USA) for 1 hour at 4^o^C After washing with 5 mM imidazole, 6×His-tagged proteins were eluted with 250 mM imidazole in the same lysis buffer. After the Ni-NTA affinity chromatography step, the protein samples were further purified by size exclusion chromatography using a HiLoad 16/600 Superdex 200 gel filtration column (GE, USA) with a running buffer containing 250 mM NaCl, 50 mM Tris-HCl pH7.5, 10% glycerol, 1 mM NaN_3_, and 5 mM 2-Mercaptoethanol). The eluted peak position was 90.5 ml, corresponding to an apparent molecular mass of ~33 kDa based on the elution behaviors of protein weight markers in the same running buffer. The ACVR1 final protein stocks were concentrated by spin-filtration to 1.5–3.0 mg ml^−1^ in 50 mM Tris HCl at pH 7.2, 250–500 mM NaCl, preserved with 10% glycerol, 5 mM β-mercaptoethanol, 1 mM sodium azide and stored at −80^o^C. Each protein sample displayed only one major band slightly above the 35 kDa molecular weight marker (Trident Prestained Protein Ladder, GeneTex, USA) on SDS-PAGE (SurePAGE 4–12% gradient gel, Genscript, USA) (Fig. 1). The absolute protein stock concentrations used in the kinase assays were quantified based on protein band density on Coomassie-stained SDS-PAGE gels compared to that of the 2 mg/ml bovine serum albumin (BSA, from Sigma, USA) standard using the ImageJ software.

K02288, quercetin, phosphoenolpyruvate, dephosphorylated bovine casein, and all of the nucleotides including ATP, ADP and NADH were obtained from Sigma, USA. The sunitinib free base form was obtained from LC Laboratories, USA. Sypro orange was obtained from Thermo Fisher Scientific, USA. Rabbit muscle pyruvate kinase and the lactate dehydrogenase PK/LDH kit were purchased from Sigma, USA. All other chemicals and reagents were purchased either from Thermo Fisher Scientific, USA or Sigma, USA unless otherwise stated.

### Kinase assays

Steady-state kinetics reactions of the catalytic amount of ACVR1 (200–300 nM) with the saturated amount of ATP substrate (0.5 mM) were carried out at either room temperature 22 ± 1^o^C or physiological temperature 37 ± 1 °C for 30 minutes. The ADP product at the end of the reaction was immediately quantified by coupled enzyme reactions of pyruvate kinase and lactate dehydrogenase (Rabbit muscle PK/LDH kit was from Sigma, USA). In particular, ADP was stoichiometrically converted to NADH whose rate was followed by UV-vis spectroscopy at 340 nm. The exact ADP amount was quantified by extrapolating the NADH oxidation initial rate to those of the ADP concentration standards in the linear range (1, 10, 25, 50, 100, 250 μM, R^2^ = 0.99). The baseline ATP hydrolysis in the absence of ACVR1 and baseline NADH oxidation in the coupled enzyme assays without addition of ADP or ATP were both near the detection limit (~5–11% of raw signal) and subtracted in each replicate to quantify the true ATP hydrolysis rate catalyzed by ACVR1. All ACVR1 kinase reactions were carried out in a kinase buffer derived from our previous protocol^29^ containing 50 mM HEPES (pH 7.3), 40 mM KCl, 75 mM NaCl, 10 mM MgCl_2_, 1% DMSO (v/v) supplemented with additional 10 mM MnCl_2_ according to *in vitro* ACVR1 kinase assays reported by other groups^18,19,27^. The coupled PK/LDH reactions were carried out in the same kinase buffer with additional 0.3 mM NADH, 3.5 mM phosphoenolpyruvate and initiated by adding 10 U/mL PK/LDH (enzyme units according to the manufacture label). The steady-state observed rate *k*_obs_ (s^−1^) was calculated by normalization with the absolute enzyme concentration. *k*_obs_ (s^−1^) measured here is estimated to be comparable to rate constant *k*_cat_ (s^−1^) because ATP is at a saturated concentration throughout the kinase reaction. In particular, [ATP] in the range of 300–500 μM within the 30 min reaction is over 10 times the previously reported *K*_m_ of wild-type (16 μM) and several mutant kinase domains including L196P, Q207E, R258S, G328E (35–40 μM)^18^, which all contain the same residues 201–499 of ACVR1 as in this study. The statistical significance between the kinetic rates of wild-type and mutant ACVR1 kinase domains were analyzed by Prism. The corresponding *p* values and number of independent replicates *n* are shown when unpaired *t*-tests identified a statistically significant difference. Statistically insignificant *p* values were not specified.

Unlike previously reported methods^18,19,27^ which use [γ-^32^P] radiolabeled ATP for kinase assays, we adopted green chemistry with coupled enzyme assays to detect ADP formation. Although *K*_m_ of the wild-type kinase domains and several other mutants have been previously reported, the concentration-normalized catalytic rate *k*_obs_ (s^−1^) and rate constant *k*_cat_ (s^−1^) have not, nor were they compared for ACVR1 wild-type or mutants to assess the differences in kinase activities. Instead, the percentage inhibition was explored, and IC50 value was often reported by other groups with an emphasis on the development of new inhibitors of ACVR1^18,19,27^. Our study represents the first report of quantifying ACVR1 kinase activity which allows for a direct comparison of the intrinsic catalytic rates of ATP hydrolysis by wild-type and mutant ACVR1.

We also note that the few previous reports of *in vitro* kinase assays of purified ACVR1 kinase domains were only carried out at room temperature^18,19,27^. We characterized the steady-state kinetics of purified ACVR1 kinase domains at both room and physiological temperatures. At 22^o^C, the total population of each ACVR1 kinase domain in this study is estimated to be entirely in the native state based on their thermal melting curves (Fig. 3a). At 37 ^o^C, considerable denaturation occurs for the G328V and to a lesser extent for the R206H and wild-type mutants (T_m_ = 35.0 ^o^C, apo form, Fig. 3a,b). The average fractions of native kinase domain populations for wild-type, R206H and G328V are estimated to be 67.15%, 56.63%, and 24.46%, respectively, based on their corresponding thermal melting curves (Fig. 3a). To account for the thermal denaturation of isolated ACVR1 kinase domains at 37 ^o^C, we performed analysis of the intrinsic kinetic rates normalized against the native enzyme concentration (Fig. 1b) as opposed to simply normalizing against the sum of native and denatured concentrations (Supplementary Fig. 3). The correction for the native population at 37^o^C allows for the measurement of the effects of the mutation on the kinetics of folded functional enzymes. In practice, we find a statistically significant increase of intrinsic kinase activity of the G328V mutant vs. wild-type, consistent with the results at 22 ^o^C (Fig. 1).

The trend of increased intrinsic kinase activity at 37 ^o^C of the G328V mutant vs. wild-type (Fig. 1b) is strengthened by including a commonly used dephosphorylated bovine casein substrate^18,19,27^ in the kinetics assays (Fig. 3c). In contrast, at 37 ^o^C, the effect of the G328V mutation relative to the wild-type kinase activity when the kinase activity is normalized by the total enzyme concentration (the sum of both native and denatured states) is statistically insignificant (Supplementary Fig. 3). This of course grossly underestimates the increase in the intrinsic kinase activity of G328V mutant vs. wild-type.

Consistent with previous reports, we found that K02288 (IC50 of 1–2 nM; *K*_d_ of 7.9 nM)^19^ at a 1μM concentration can completely abolish the kinase activity of the wild-type, R206H and G328V mutant ACVR1 kinase domains (Fig. 1).

### Thermal shift assays

Briefly, thermal denaturation assays of the proteins were performed in 96-well plates using a RealPlex quantitative PCR instrument from Eppendorf (Eppendorf, USA). The system was automatically precalibrated before each run. Then, 20 µL of protein samples, each at a fixed concentration in the range of 1–2 µM, were aliquoted into the bottom of the wells, incubated with the compounds of interest for 10–30 min at room temperature, and mixed with SYPRO orange (serially diluted to a final concentration of 5× from a 5000× stock solution). The ACVR1 kinase domain stocks were thawed on ice and extensively buffer-exchanged into 50 mM HEPES pH 7.3, 100 mM NaCl at 4 ^o^C by Amicon® Ultra-4 Centrifugal Filter Units with a 10 kDa molecular weight cutoff (Millipore, USA). The buffer-exchange step resulted in an ~1000-fold dilution of the initial buffer content in the original protein stock. This avoids possible stabilizing effects due to residual glycerol content across wild-type, R206H and G328V ACVR kinase domains in the thermal shift assays and allows for a more reliable comparison of the relative thermal stability of different protein samples. For example, the apo form of wild-type protein without a buffer-exchange step (T_m_ = 40.8 ± 0.6 ^o^C) appeared to be slightly more stable than the buffer-exchanged, wild-type protein (T_m_ = 38.5 ± 0.6 ^o^C) (Supplementary Fig. 2a vs. Supplementary Fig. 1a). The same reaction buffer containing 50 mM HEPES pH 7.3, 100 mM NaCl was used for all the thermal shift assays unless otherwise stated. A heating ramp of 1 °C/min from 20 °C to 80 °C was used, and one data point acquired for each degree increment. The excitation and emission wavelengths were 465 and 580 nm, respectively. The compound/buffer control melting curve was subtracted from the samples containing the protein of interest. In this study, the compounds themselves typically yielded minimal fluorescence signal compared to the protein’s melting curve. The negative first derivative of relative fluorescence with arbitrary units, −d(FAU)/dt, was plotted against T with Excel software to determine the melting temperature T_m_. Only one negative peak in the plot was observed for each protein sample, indicating concerted melting of the enzyme following a quasi-two-state model. The thermal shift ∆T_m_ = T_m_ (protein with drug) – T_m_ (protein). Each condition was run at least in duplicate. The corresponding *p* values were shown when unpaired *t*-tests by Prism identified stringent statistical significance. All melting curves, thermal shift and kinetics assays plots were generated with the Prism software. To estimate a ligand binding affinity to a protein, thermal shift assays were performed for a series of ligand concentrations (Supplementary Fig. 2). The dose-dependence of thermal shifts allows a linear fitting of ln[ligand] vs. 4.184 × 1000(1/T_0_ − 1/T_m_)/R where T_0_ is the melting temperature of the apo protein in the absence of ligand of interest, and T_m_ is melting temperature at a given ligand concentration. Consequently, the ligand affinity can be obtained as *K*_d_ (μM) ≈ e^y-intercept^, an approximation under conditions of [ligand] ≫ [protein] during the thermal transition based on previously derived binding thermodynamics equations^29,43,44^.

### Off-targets predictions by FINDSITE^comb2.0^ virtual target screening

The prediction results are summarized in Supplementary Table 1. The mTC virtual screening score is used to rank the targets from FINDSITE^comb2.0^ predictions^29^. The mTC score is a measure of the similarity of the given drug to the template ligands of a protein target. It correlates with the likelihood of the protein target being a true target of the drug and its value is between 0 and 1 (with 1 being the best). We list targets with an mTC > 0.5 (corresponding to expected precision of ~0.76 based on benchmark statistics), except for LDN214117 for which the cutoff is set near ~0.488 (corresponding to expected precision of 0.65) to show its known true on-target ACVR1. Predictions of the number of tissue sites for which a gene is a potential cancer driver are listed^45–47^ (see below). The mTC score correlates with the expected precision of predicted off-target interactions but does not suggest a mode of action (agonist vs. antagonist). TGF-β Ser/Thr receptor kinases family are highlighted as yellow (type II, 5 members) and cyan (type I, ALK1,2,3,4,5,6,7). *ACVR1 (ALK2, labeled red) is also included as a reference for true on-target.

To examine the importance of a particular target gene to a given cancer, we examined its somatic mutations in the COSMIC database (cancer.sanger.ac.uk/cosmic) using ENTPRISE or ENTPRISE-X^45,46^. A gene is a potential cancer driver when at least one somatic mutation in the COSMIC database is predicted to be disease associated by ENTPRISE or ENTPRISE-X. The COSMIC database also provides the tissue site of the cancers and mutations. There are total 42 tissue sites in the COSMIC database including *bone, breast, central nervous system, cervix, ovary, prostate, skin, etc*. Thus, we can infer how many cancer tissue sites a gene could potentially affect. For example, POMK could be cancer driver of *breast, large intestine, liver and stomach*.

### DIPG cell culture and viability assay

The SF8628 human patient derived DIPG cell line harboring H3.3K27M histone mutation was purchased from MilliporeSigma, USA. The TβRI inhibitors EW7197 (vactosertib), LY3200882 and LY2157299 (galunisertib), SB525334, histone deacetylase inhibitor panobinostat and histone demethylase inhibitor GSK-J4 (all from Fisher Scientific, USA and manufactured by Cayman Chemicals) were administered as drug treatments to assess their effects on SF8628 cell growth. Cell culture stocks frozen in DMEM high glucose media (Fisher Catalog #11965118) supplemented with 10% premium fetal bovine serum FBS (Fisher Catalog # MT35015CV) and 1X Penicillin-Streptomycin antibiotics (Fisher Catalog #15140122) and containing 10% DMSO were stored in liquid nitrogen. After being thawed, the cells were cultured in the same DMEM media mixture without DMSO at 37 °C in a 5% CO_2_ atmosphere in Corning T75 culture flasks (Fisher Catalog # 07202000) and passaged upon near confluence following manufacture protocol of MilliporeSigma, USA.

The SU-DIPG-IV DIPG cell line harboring the H3.1K27M histone and ACVR1 G328V mutations was acquired from Dr. Michelle Monje at Stanford University. Cells were cultured in either DMEM media the same way as in SF8628 cells or in tumor stem media (TSM) derived from previous SU-DIPG-IV studies^12^ supplemented with B-27 serum-free supplement minus vitamin A (Thermal Fisher Catalog #12587010), human EGF (Shenandoah Biotech Catalog # 100-26), FGF (Shenandoah Biotech Catalog # 100-146), PDGF-AA (Shenandoah Biotech Catalog # 100-16) and PDGF-BB (Shenandoah Biotech Catalog # 100-18) growth factors and heparin (StemCell Technologies, Catalog # 07980) except that 1X Penicillin-Streptomycin antibiotics (same as in DMEM media) instead of Antibiotic-Antimycotic were used. This permits a comparison between different media conditions without complications from different antimicrobial reagents.

To set up cell cultures for the Tox-8 assays^38^, the cells were applied onto a Corning™ Falcon™ 96-Well Imaging Microplate (Corning Ref# 353219), with 100 µL containing 2,000 cells in the culturing media per each well. After 24 hours of incubation at 37 ^o^C in a 5% CO_2_ atmosphere, the cells were treated with a series of concentrations of the drug (single agent or combinations) of interest vs. DMSO control by adding 100 µL of 2x drug stocks or the corresponding DMSO control prepared in the same media. Each concentration condition had three to four replicates. After 72 hours of drug treatment, the cells were analyzed for their viability using the Tox-8 assay kit (Sigma, USA) following the manufacturer’s protocol. In particular, 20 µL of Tox-8 solution was added to each well and the metabolic activity of the living cells was assessed based on their bioreduction of the exogenously introduced Resazurin fluorescent dye. The fluorescent signals with excitation and emission wavelengths at 560 nm and 590 nm were recorded at 3 h after introducing the dye using a BioTek Synergy4 fluorescence microplate reader following Tox8 assay protocols according to vendors protocol Sigma, USA. Dose response curve and IC50 analysis was performed with Prism8 software using default three parameters drug inhibition curve fitting.

## Supplementary information

Supplementary information.

## Data Availability

All data supporting this study are provided and available from the authors upon reasonable request.
