# Supplementary material for: Differential kinase activity of ACVR1 G328V and R206H mutations with implications to possible TβRI cross-talk in diffuse intrinsic pontine glioma
Source: Sci Rep. 2020 Apr 9;10:6140. doi: 10.1038/s41598-020-63061-0 (PMC7145857; doi:10.1038/s41598-020-63061-0)
Supplement: Supplementary file 1 — Supplementary information. [file 41598_2020_63061_MOESM1_ESM.pdf]

Supplementary Information

Differential kinase activity of ACVR1 G328V and R206H mutations with implications to possible TβRI cross-talk in diffuse intrinsic pontine glioma

By

Hongnan Cao[1], Miao Jin[2], Mu Gao[1], Hongyi Zhou[1], Yizhi Jane Tao[2] and Jeffrey Skolnick[1,*]

[1]Center for the Study of Systems Biology, School of Biological Sciences, Georgia Institute of Technology, 950 Atlantic Drive, NW, Atlanta, Georgia 30332, United States.

[2]Department of BioSciences, Rice University, Houston, Texas 77005, United States.

***E-mail: skolnick@gatech.edu**

Tel: (404) 407-8975

Fax: (404) 385-7478

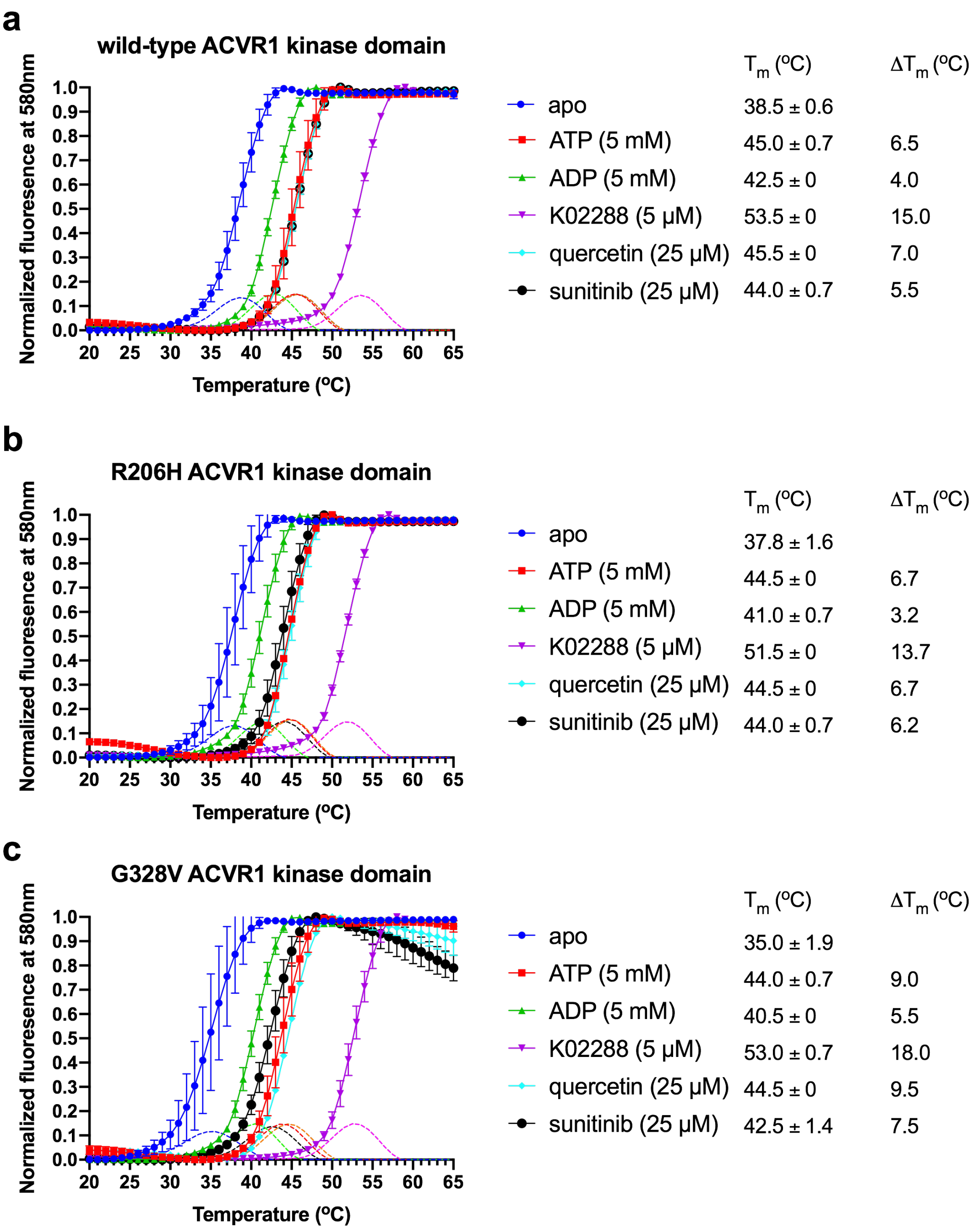

Fig. S1. Thermal shift assay melting curves of wild-type (a), R206H (b) and G328V (c) ACVR1 kinase domains. The slope of each curve is also plotted as dotted lines with the corresponding color coding. ACVR1 kinase domains were extensively buffer exchanged into 50 mM HEPES, 100 mM NaCl, pH 7.3 before the thermal shift assay was performed (see Methods in the main text).

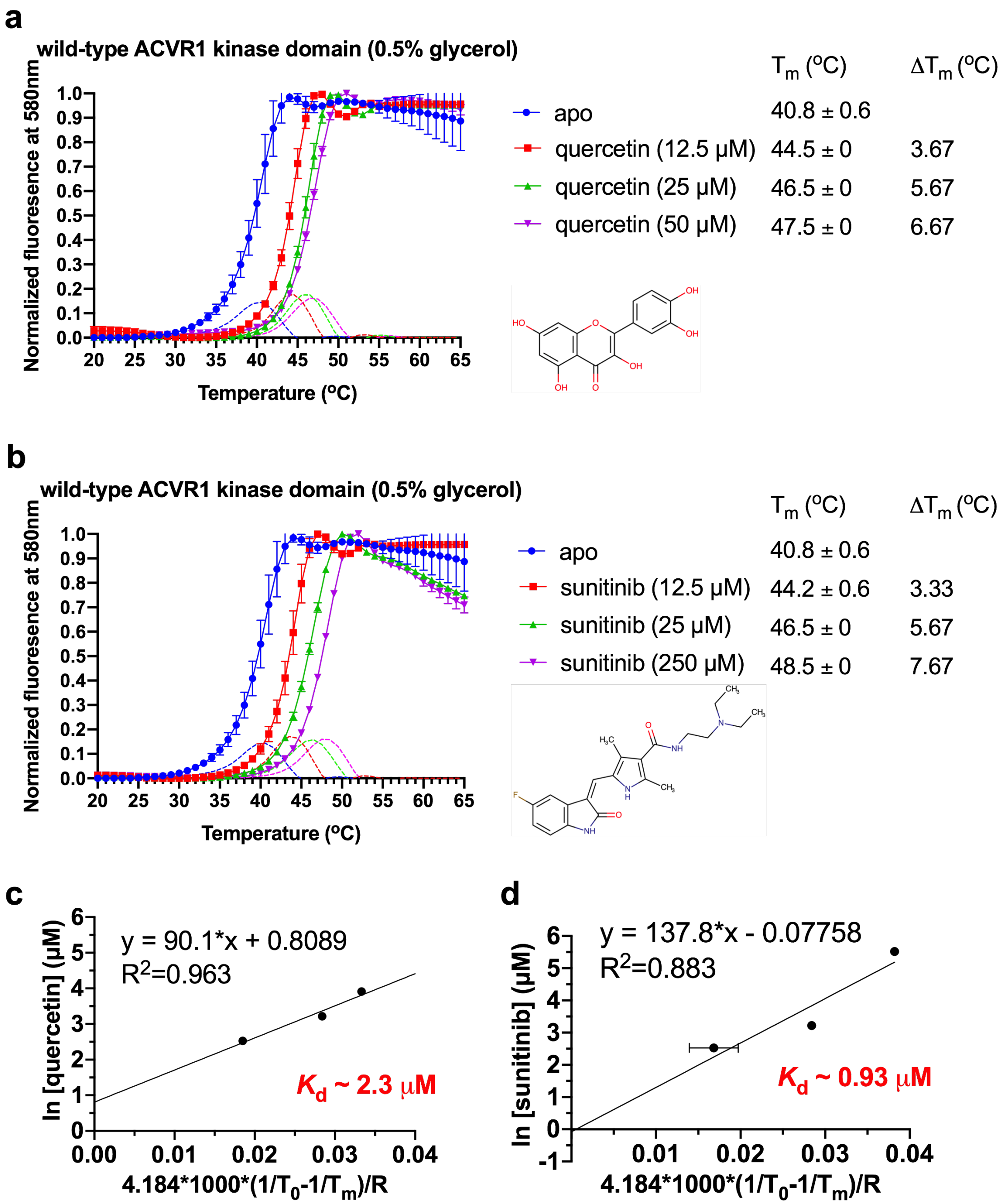

Fig. S2. Thermal shift assays of wild-type ACVR1 kinase domains with quercetin (a) and sunitinib (b). The slope of each curve is also plotted as dotted lines with the corresponding color coding. The 2D chemical structures of quercetin (a, right) and sunitinib (b, right) are also shown. ACVR1 kinase domains were diluted without extensive buffer exchange and contained 0.5% glycerol in final reaction mix solutions. Also shown is a linear regression plot of ln [ligand] against $4.184*1000*(1/T_o-1/T_m)/R$ to estimate the ligand's K_d for quercetin (c) and sunitinib (d) (see Methods in the main text).

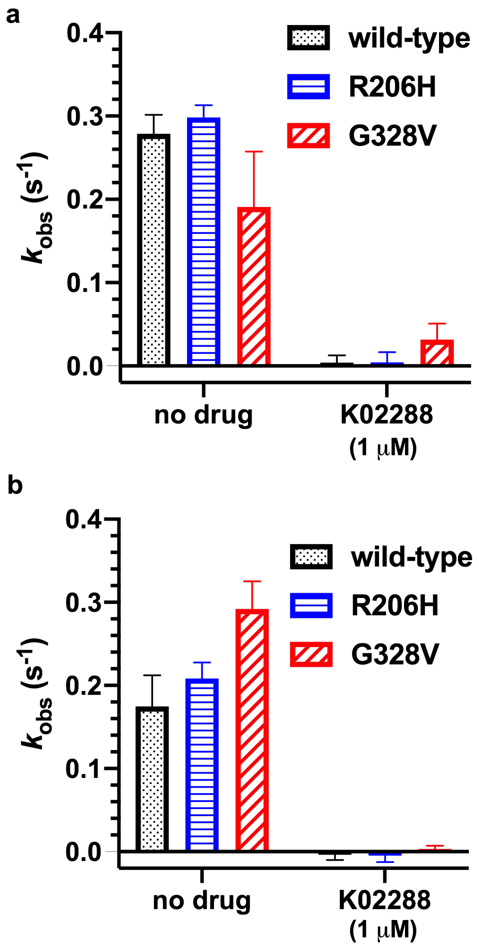

Fig. S3. The intrinsic ATPase activity wild-type, R206H and G328V mutants of ACVR1 kinase domains with 0.5mM ATP in pH 7.3 kinase buffer at 37°C without (a) or with (b) 0.5 mg/ml dephosphorylated casein substrate. The turnover rate k_{obs} is quantified based on the ADP and normalized by the total (sum of native and denatured) ACVR1 concentrations. Based on unpaired *t*-test, there is no statistically significance observed when comparing G328V and wild-type ACVR1 activities at 37°C when normalizing k_{obs} against the total enzyme concentration as opposed to correction for the native enzyme concentration (main text, Figs. 1b, 3c).

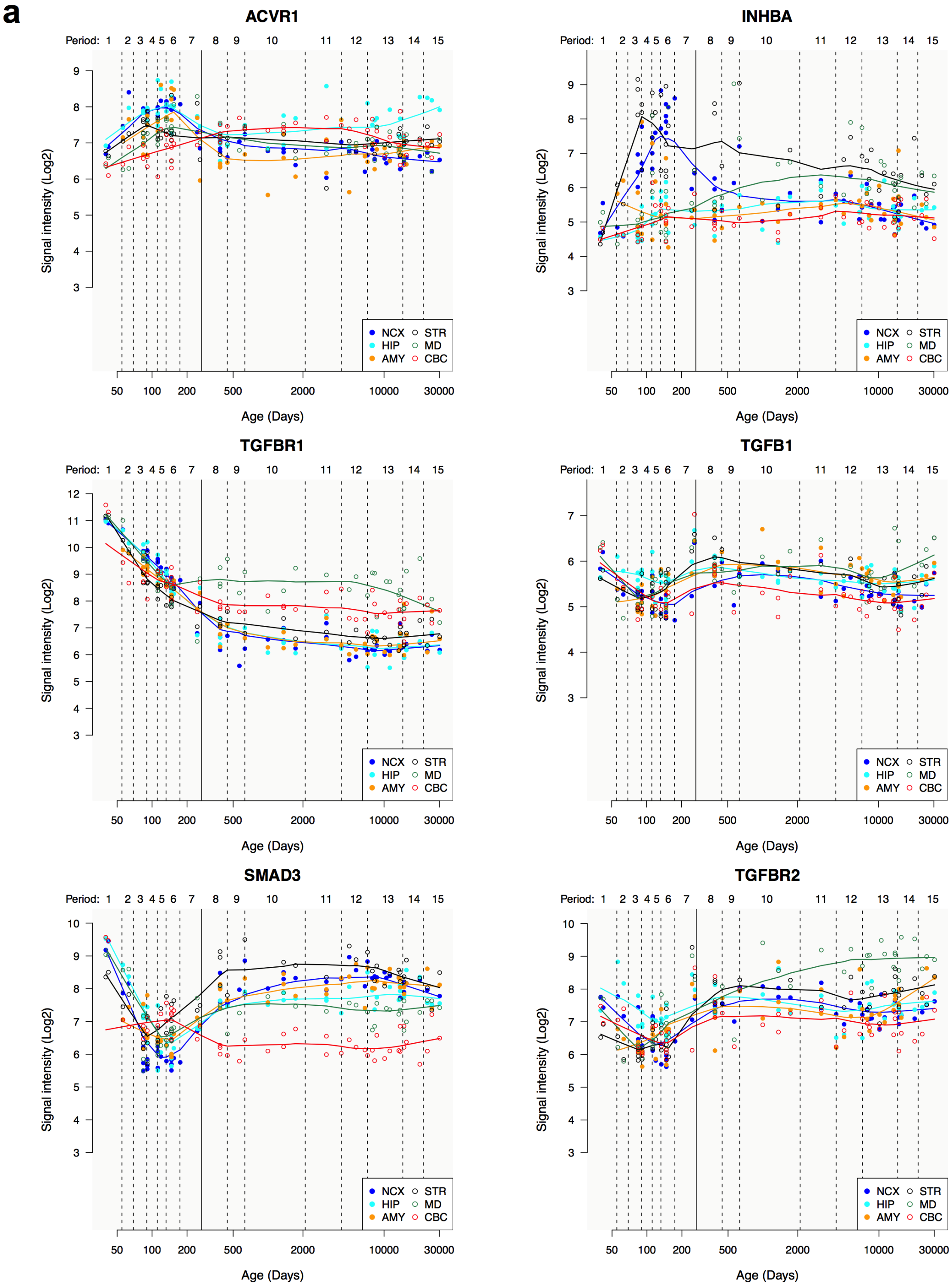

a
ACVR1
INHBA
TGFBR1
TGFB1
SMAD3
TGFBR2
Period: 1 2 3 4 5 6 7 8 9 10 11 12 13 14 15
Signal intensity (Log2)
Age (Days)
NCX STR
HIP MD
AMY CBC

b

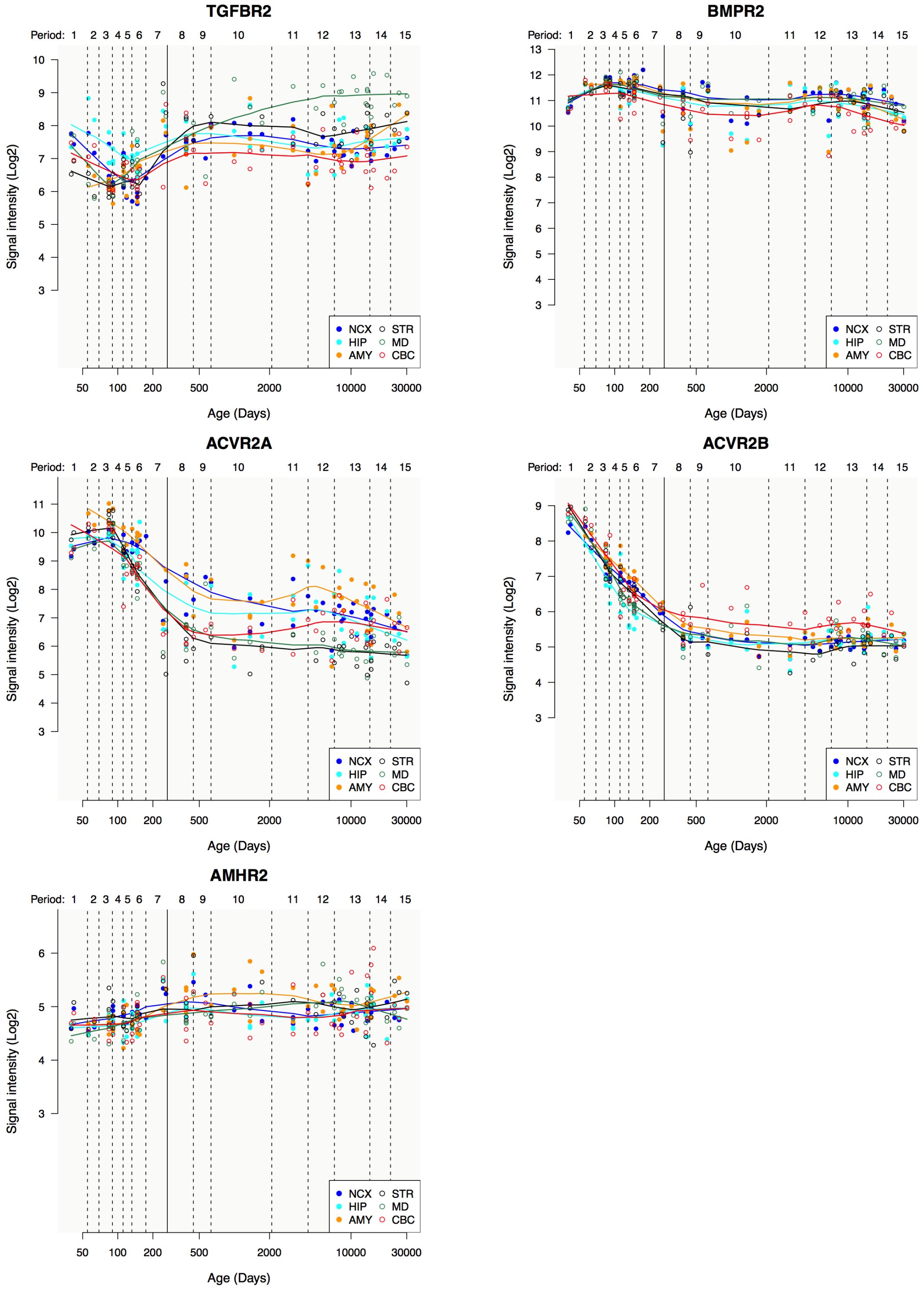
TGFBR2
BMPR2
ACVR2A
ACVR2B
AMHR2
Period: 1 2 3 4 5 6 7 8 9 10 11 12 13 14 15
Signal intensity (Log2)
Age (Days)
50 100 200 500 2000 10000 30000
NCX
HIP
AMY
STR
MD
CBC

c

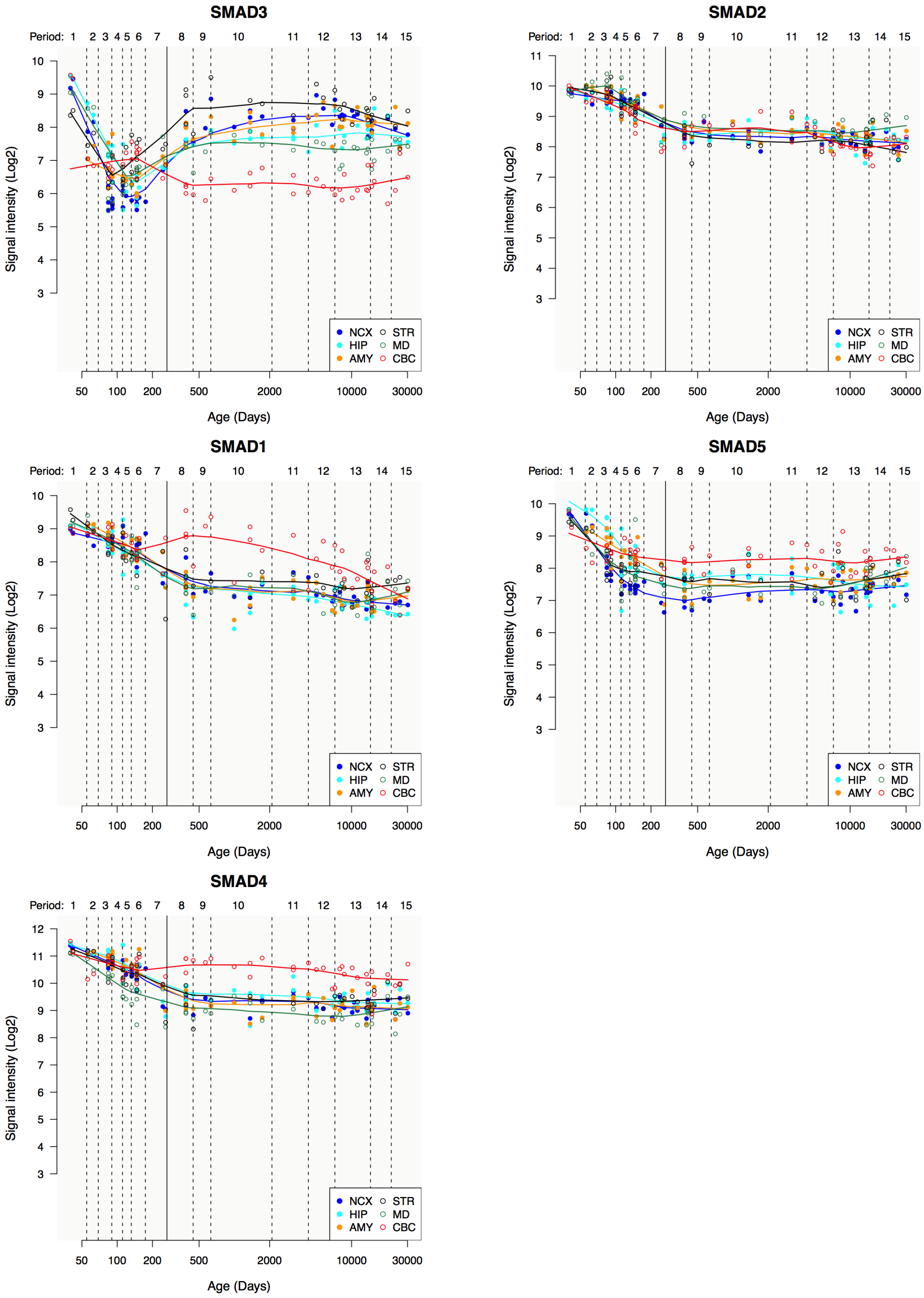
SMAD3
SMAD2
SMAD1
SMAD5
SMAD4
Period: 1 2 3 4 5 6 7 8 9 10 11 12 13 14 15
Signal intensity (Log2)
Age (Days)
50 100 200 500 2000 10000 30000
NCX
HIP
AMY
STR
MD
CBC

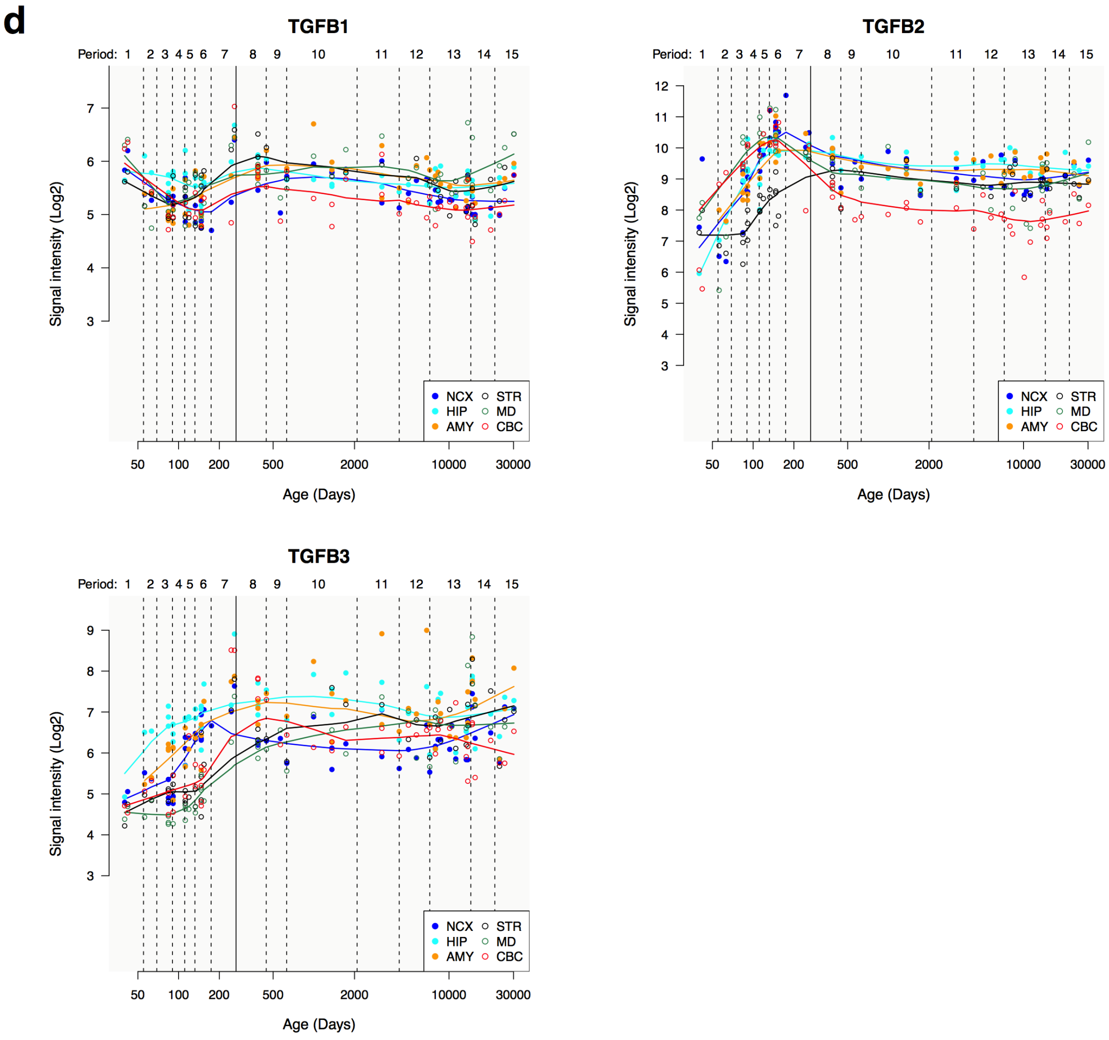

Fig. S4. Expression of TβRI, ACVR1 and their immediate upstream or downstream signaling protein partners measured as human brain transcriptome in different anatomical structures along the entire time range from embryonic development to adulthood. All the plots under analysis were obtained from the Human Brain Transcriptome (hbatlas.org). Abbreviations for brain structures: NCX, neocortex; STR, striatum; HIP, hippocampus; MD, thalamus; AMY, amygdala; CBC, cerebellar cortex. Developmental timeline by weeks: period 3, 10-13 PCW (postconceptional weeks); period 4, 13-16 PCW; period 5, 16-19 PCW, period 6, 19-24 PCW. K27M mutation expression was reported to peak spatiotemporally in the striatum (STR) and thalamus (MD) during the mid-fetal period of brain development (~19 PCW or 133 days within the periods 4,5,6). Gene (protein) names: ACVR1 (ACVR1), INHBA (activin A), TGFBR1 (TβRI), TGFB1 (TGF-β1), TGFB2 (TGF-β2), TGFB3 (TGF-β3), SMAD1 (Smad1), SMAD2 (Smad2), SMAD3 (Smad3), SMAD4 (Smad4), SMAD5 (Smad5), TGFBR2 (TβRII), BMPR2 (BMPRII), ACVR2A (ActRIIA), ACVR2B (ActRIIB), AMHR2 (AMHRII). (a) Contrast of gene expression peak increase (ACVR1) and peak decrease (TβRI) resembles the opposite trends of their key protein partners. Further comparison of expressions of (b) 5 members of type II receptors, (c) Smad 1,2,3,4,5, (d) TGF-β1,2,3.

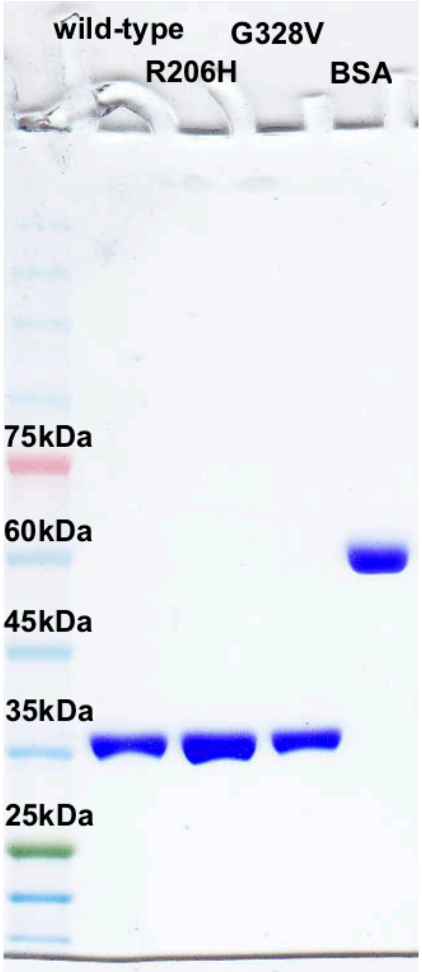

Fig. S5. Full-length gel image corresponding to the same gel lanes as in Fig. 1c in the main text. In the main text Fig. 1c, the gel image is slightly cropped from the top (loading wells) and the bottom for the purpose of conciseness. Both Fig. S5 and the corresponding Fig. 1c are the original color images without any contrast adjustment.

Table S1. Potential human off-targets of ACVR1 inhibitors predicted by FINDSITE$^{comb2.0}$ virtual target screening

**LDN193189 (CAS # 1062368-24-4)**				
Rank	mTC score	Gene ID	Gene Name	# cancer driver sites
1	0.568240	NP_001158162.1	AMHR2 (AMHRII)	11
2	0.566698	NP_001014795.1	ILK	1
3	0.566632	NP_004320.2	BMPR1A (ALK3)	10
4	0.565979	NP_001194.1	BMPR1B (ALK6)	11
5	0.558836	NP_115613.1	POMK	4
6	0.558576	NP_064732.3	ACVR1B (ALK4)	14
7	0.557908	NP_001104501.1	ACVR1C (ALK7)	12
8	0.556890	NP_002437.2	MAP3K10	8
9	0.556655	NP_001070869.1	ACVRL1 (ALK1)	13
10	0.556649	NP_001607.1	ACVR2A (ActRIIA)	19
11	0.556629	NP_001097.2	ACVR2B (ActRIIB)	4
12	0.556317	NP_004603.1	TGFBR1 (ALK5)	18
13	0.555412	NP_001104537.1	ACVR1 (ALK2)*	9
14	0.551663	NP_009130.2	IRAK3	4
15	0.550454	NP_001020018.1	TGFBR2 (TβRII)	14
16	0.549702	NP_057737.2	ZAK	3
17	0.549540	NP_612379.2	PKDCC	3
18	0.548305	NP_006862.2	RIPK3	1
19	0.546337	NP_002410.1	MAP3K11	1
20	0.543984	NP_001020414.1	IRAK1	12
21	0.542177	NP_001138864.1	TSEN2	4
22	0.541537	NP_002022.1	FRK	8
23	0.539438	NP_001195.2	BMPR2 (BMPRII)	14
24	0.538193	NP_001561.3	IRAK2	2
25	0.513279	NP_001037834.2	FAM69C	3
26	0.507441	NP_067585.2	BCR	10
27	0.507441	NP_009297.2	ABL1	14
28	0.507441	NP_054700.2	DDR1	12
29	0.507441	NP_001014796.1	DDR2	11
30	0.501145	NP_005363.1	MOS	7
**LDN212854 (CAS # 1432597-26-6)**				
Rank	mTC score	Gene ID	Gene Name	# cancer driver sites
1	0.566540	NP_001158162.1	AMHR2 (AMHRII)	11
2	0.564586	NP_001014795.1	ILK	1
3	0.564382	NP_004320.2	BMPR1A (ALK3)	10
4	0.563726	NP_001194.1	BMPR1B (ALK6)	11

5	0.556506	NP_115613.1	POMK	4
6	0.556394	NP_064732.3	ACVR1B (ALK4)	14
7	0.555750	NP_001104501.1	ACVR1C (ALK7)	12
8	0.554912	NP_002437.2	MAP3K10	8
9	0.554707	NP_001607.1	ACVR2A (ActRIIA)	19
10	0.554687	NP_001097.2	ACVR2B (ActRIIB)	4
11	0.554421	NP_001070869.1	ACVRL1 (ALK1)	13
12	0.554132	NP_004603.1	TGFBR1 (ALK5)	18
13	0.553260	NP_001104537.1	ACVR1 (ALK2)*	9
14	0.549599	NP_009130.2	IRAK3	4
15	0.548502	NP_001020018.1	TGFBR2 (TβRII)	14
16	0.547920	NP_057737.2	ZAK	3
17	0.547340	NP_612379.2	PKDCC	3
18	0.546285	NP_006862.2	RIPK3	1
19	0.544770	NP_002410.1	MAP3K11	1
20	0.541997	NP_001020414.1	IRAK1	12
21	0.540395	NP_001138864.1	TSEN2	4
22	0.539434	NP_002022.1	FRK	8
23	0.537828	NP_001195.2	BMPR2 (BMPRII)	14
24	0.536315	NP_001561.3	IRAK2	2
25	0.511862	NP_001037834.2	FAM69C	3
26	0.505252	NP_067585.2	BCR	10
27	0.505252	NP_009297.2	ABL1	14
28	0.505252	NP_054700.2	DDR1	12
29	0.505252	NP_001014796.1	DDR2	11
30	0.500102	NP_005363.1	MOS	7
**LDN214117 (CAS # 1627503-67-6)**				
Rank	mTC score	Gene ID	Gene Name	# cancer driver sites
1	0.536511	NP_001032827.1	NPM1	4
2	0.513456	NP_001137276.1	PHOSPHO1	3
3	0.513456	NP_878911.1	PRDM1	11
4	0.507199	NP_001158162.1	AMHR2 (AMHRII)	11
5	0.503218	NP_001014795.1	ILK	1
6	0.502464	NP_002410.1	MAP3K11	1
7	0.502454	NP_002437.2	MAP3K10	8
8	0.499299	NP_006862.2	RIPK3	1
9	0.497082	NP_004320.2	BMPR1A (ALK3)	10
10	0.496789	NP_149132.2	MAP3K9	5
11	0.496397	NP_001194.1	BMPR1B (ALK6)	11

12	0.496216	NP_612379.2	PKDCC	3
13	0.495510	NP_057737.2	ZAK	3
14	0.494023	NP_001607.1	ACVR2A (ActRIIA)	19
15	0.493933	NP_001097.2	ACVR2B (ActRIIB)	4
16	0.493196	NP_064732.3	ACVR1B (ALK4)	14
17	0.493058	NP_001020018.1	TGFBR2 (TβRII)	14
18	0.492798	NP_115613.1	POMK	4
19	0.491953	NP_001104503.1	ACVR1C (ALK7)	12
20	0.491219	NP_009130.2	IRAK3	4
21	0.491145	NP_001973.2	ERBB3	13
22	0.490906	NP_001020414.1	IRAK1	12
23	0.490130	NP_004603.1	TGFBR1 (ALK5)	18
24	0.489219	NP_001070869.1	ACVRL1 (ALK1)	13
25	0.488738	NP_001104537.1	ACVR1 (ALK2)*	9
26	0.487749	NP_001195.2	BMPR2 (BMPRII)	14
**K02288 (CAS # 1431985-92-0)**				
Rank	mTC score	Gene ID	Gene Name	# cancer driver sites
1	0.522745	NP_002603.1	PDK4	9
2	0.522745	NP_004472.1	GALNT2	2
3	0.515163	NP_001158162.1	AMHR2 (AMHRII)	11
4	0.512140	NP_002410.1	MAP3K11	1
5	0.510234	NP_001014795.1	ILK	1
6	0.509378	NP_004320.2	BMPR1A (ALK3)	10
7	0.508653	NP_001194.1	BMPR1B (ALK6)	11
8	0.506476	NP_006862.2	RIPK3	1
9	0.505846	NP_009130.2	IRAK3	4
10	0.505663	NP_064732.3	ACVR1B (ALK4)	14
11	0.504840	NP_001607.1	ACVR2A (ActRIIA)	19
12	0.504756	NP_115613.1	POMK	4
13	0.504367	NP_612379.2	PKDCC	3
14	0.504365	NP_001097.2	ACVR2B (ActRIIB)	4
15	0.504307	NP_001104501.1	ACVR1C (ALK7)	12
16	0.503928	NP_004603.1	TGFBR1 (ALK5)	18
17	0.502991	NP_001020018.1	TGFBR2 (TβRII)	14
18	0.502917	NP_001138864.1	TSEN2	4
19	0.502629	NP_001070869.1	ACVRL1 (ALK1)	13
20	0.502452	NP_001104537.1	ACVR1 (ALK2)*	9
21	0.500586	NP_001137276.1	PHOSPHO1	3
22	0.500586	NP_878911.1	PRDM1	11

TGF-β Ser/Thr receptor kinases family are highlighted as yellow (type II, 5 members) and cyan (type I, ALK1,2,3,4,5,6,7). *ACVR1 (ALK2, labeled red) is also included as a reference for a true on-target. The details of prediction procedures are described in Methods in the main text.
